# A single immunization with HA DNA vaccine by electroporation induces early protection against H5N1 avian influenza virus challenge in mice

**DOI:** 10.1186/1471-2334-9-17

**Published:** 2009-02-12

**Authors:** Liyun Zheng, Fuyan Wang, Zhongdong Yang, Jianjun Chen, Haiyan Chang, Ze Chen

**Affiliations:** 1 State Key Laboratory of Virology, Wuhan Institute of Virology, Chinese Academy of Sciences, Wuhan 430071, PR China; 2 Graduate University of Chinese Academy of Sciences, Beijing 100049, PR China; 3 College of Life Science, Hunan Normal University, Changsha 410081, Hunan, PR China; 4 Shanghai Institute of Biological Products, Shanghai 200052, PR China

## Abstract

**Background:**

Developing vaccines for the prevention of human infection by H5N1 influenza viruses is an urgent task. DNA vaccines are a novel alternative to conventional vaccines and should contribute to the prophylaxis of emerging H5N1 virus. In this study, we assessed whether a single immunization with plasmid DNA expressing H5N1 hemagglutinin (HA) could provide early protection against lethal challenge in a mouse model.

**Methods:**

Mice were immunized once with HA DNA at 3, 5, 7 days before a lethal challenge. The survival rate, virus titer in the lungs and change of body weight were assayed to evaluate the protective abilities of the vaccine. To test the humoral immune response induced by HA DNA, serum samples were collected through the eye canthus of mice on various days after immunization and examined for specific antibodies by ELISA and an HI assay. Splenocytes were isolated after the immunization to determine the antigen-specific T-cell response by the ELISPOT assay.

**Results:**

Challenge experiments revealed that a single immunization of H5N1 virus HA DNA is effective in early protection against lethal homologous virus. Immunological analysis showed that an antigen-specific antibody and T-cell response could be elicited in mice shortly after the immunization. The protective abilities were correlated with the amount of injected DNA and the length of time after vaccination.

**Conclusion:**

A single immunization of 100 μg H5 HA DNA vaccine combined with electroporation was able to provide early protection in mice against homologous virus infection.

## Background

The outbreak of human infections of H5N1 influenza in 1997 in Hong Kong and in 2003–2004 in most Asian countries demonstrated that purely avian viruses could be transmitted to humans and cause severe disease [1]. Prior to the Hong Kong outbreak, H5 influenza viruses had been isolated only from avian species [2]. They exist in a non-pathogenic form in wild aquatic birds in different regions of the world and in domestic ducks in Southern China [2-4]. Highly pathogenic avian influenza (HPAI) H5N1 viruses are now enzootic in several countries and are presently undergoing unprecedented geographic expansion among wild and domestic birds [1,5-8]. Some person-to-person transmissions in family clusters have been observed in Vietnam, Thailand and Indonesia [9-11]. Although all H5N1 viruses isolated from humans retain characteristic features of avian influenza viruses and are not currently transmissible among humans, the potential for a pandemic caused by H5N1-HPAIV is increasing [8,12].

To prevent influenza, a protective immunity must be induced, in advance, by vaccination. Immunization with inactivated vaccines has been the main technique used to prevent avian influenza for a long time. Some studies demonstrated that inactivated H5 vaccines could protect chickens and mice against the challenge with the homologous virus [7,13,14]. Meanwhile, it has been reported that immunizations with avian influenza H5N1 inactivated vaccines induced protective antibodies in humans [5,15,16]. Immunization with DNA vaccines is also one of the strategies for preventing avian influenza. Many studies showed that DNA vaccines could provide protection for chickens and mice against avian influenza types H3, H5, H7 and H9 [7,17-22]. Our previous studies also showed that both hemagglutinin (HA)- and neuraminidase (NA)-DNA vaccines could protect mice from the challenge with either influenza A or B viruses [23-28].

In this study, an avian influenza virus strain A/Chicken/Henan/12/2004 (H5N1) was isolated from a farmed chicken in Henan province, China. The H5 virus was found to be able to replicate in BALB/c mice without adaptation and caused mortality, which demonstrated the infectivity of influenza H5N1 virus among species. The HA gene was cloned from the virus and the abilities of an HA DNA vaccine to provide protection for BALB/c mice against homologous virus infection were explored. We showed that a single immunization of H5N1 DNA vaccine was able to provide early protection in mice against homologous virus infection.

## Methods

### Virus

The virus A/Chicken/Henan/12/2004(H5N1) was isolated from a farmed chicken in Henan province, China. Viral isolates were identified by the hemagglutination assay after inoculating the allantoic cavity of 10 day-old specific pathogen-free (SPF) chicken embryos. Three days after the inoculation, allantoic fluids from infected eggs were harvested, aliquoted and stored in at -80°C. The 50% embryo lethal dose (ELD_50_) was determined for each stock and the viruses were subsequently isolated in a Biosafety Level 3 (BSL-3) facility. The viral RNA from the isolates propagated in 10-day-embryonated eggs was extracted by the cleavage of viruses with Trizol LS Reagent (Life Technologies, Inc.). The RNA was reverse-transcribed into single-stranded cDNA with a first strand cDNA synthesis kit (AMV) (Roche Diagnostics). The viral HA gene was amplified by PCR using the Expand High Fidelity PCR System (Roche Diagnostics) with virus-specific primers (F Primer 5'-GGT**CTCGAG**TGTCAAA**ATG**GAGAAAATAGTGCTT-3', *Xho*I site and start codon in bold; R Primer, 5'-TCT**CCCGGG**ACAAAT**TTA**AAT GCAAATTCTGCAT-3', *Sma *I site and stop codon in bold), then sequenced by the dideoxy method using an ABI PRISM 377 DNA Sequencer (Applied Biosystems).

The virus was found to be able to directly replicate in BALB/c mice without adaptation and caused mortality (data not shown). To prepare adapted virus to mice, lung-to-lung passages were performed. Briefly, the BALB/c mice were anesthetized and inoculated with 20 μl of the above-mentioned H5N1 viral suspension by intranasal drip. At the 3rd day after inoculation, the mice were sacrificed, and their trachea and lungs were taken out and washed 3 times with a total of 2 ml of phosphate buffered saline (PBS) containing 0.1% bovine serum albumin (BSA). The bronchoalveolar washings were collected and used for infecting the next batch of mice after removing the cellular debris by centrifugation [28]. The lung-to-lung passages were repeated at least three times and the adapted viruses were harvested, aliquoted, and stored in at -80°C. The 50% mouse lethal dose (MLD_50_) of each stock was determined using the Reed-Muench method [29].

### Plasmid DNAs

The plasmid pCAGGSP7/HA (HA DNA) was constructed by cloning the PCR product of the HA gene from A/Chicken/Henan/12/2004 (H5N1) influenza virus strain into the expression vector pCAGGSP7, as described previously [23,30]. The nucleotide sequence of the HA gene was confirmed by the dideoxy method using ABI PRISM 377 DNA Sequencer (Applied Biosystems). The plasmid was propagated in Escherichia coli XL1-blue bacteria and purified using QIAGEN Purification Kits (QIAGEN-tip 500).

### Nucleotide sequence accession numbers

The HA gene sequence from A/Chicken/Henan/12/2004 (H5N1) has been deposited in GenBank. The accession number is AY950232.

### Immunization by in vivo electroporation

For immunization, the BALB/c mice were anaesthetized with a mixture of ketamine and lobelanine and injected with HA DNA into the right quadriceps muscle. A pair of 5-mm-apart electrode needles (26-gauge) was then inserted into the muscle to cover the DNA injection sites and electrical pulses were delivered using an electric pulse generator (Electro Square Porator T830 M; BTX, San Diego, CA). Three pulses of 100 V each were delivered to the injection site at a rate of one pulse per second, each lasting for 50 ms [31]. Three pulses of the opposite polarity were then applied. All procedures described above have been reviewed and approved by Animal Care Committee of Wuhan Institute of Virology, Chinese Academy of Sciences.

### Infection

The mice were anesthetized and challenged with the mouse-adapted strain A/Chicken/Henan/12/2004(H5N1) (5 LD_50_) by intranasal administration with 20 μl of the viral suspension at 3, 5 or 7 days after immunization. This infection caused rapid and widespread viral replication in the lung and death of the control non-immunized mice within 7–10 days [32].

### Specimens

Blood samples were collected from either medial or lateral canthus of mice on various days after immunization for antibody detection. For lung virus titration, mice were sacrificed three days after the viral challenge. A ventral incision was made along the median line from the xiphoid process to the point of the chin. The trachea and lungs were taken out and washed twice by injecting 2 ml of PBS containing 0.1% BSA. The bronchoalveolar wash was used for virus titration after removing cellular debris by centrifugation [27].

### Antibody detection by ELISA and HI assay

The serum IgM and IgG Abs against HA were measured by ELISA. ELISA was performed sequentially in a 96-well polystyrene microtiter plate with reagents consisting of: 1) HA molecules purified from the H5N1 virus [33]; 2) serial two-fold dilutions of sera from mice; 3) goat anti-mouse IgG or IgM Ab (Southern Biotechnology Associates, Inc. USA) conjugated with biotin; 4) streptavidin conjugated with alkaline phosphatase (Southern Biotechnology Associates, Inc. USA); and 5) *p*-nitrophenyl-phosphate. The amount of chromogen produced was measured based on absorbance at 414 and 405 nm in a Labsystems Multiskan Ascent Autoreader (model 354, Finland). The Ab-positive cut-off values were set as mean + 2 SD of non-immunized sera. An ELISA Ab titer was expressed as the highest serum dilution giving a positive reaction.

The serum neutralization activity was measured by hemagglutination inhibition assays[34]. Receptor destroying enzyme (RDE)-treated sera were serially diluted (2 fold) in V-bottom 96-well plates. Approximately 4 HA units of viral antigen was incubated with the serum for 30 min at room temperature, followed by the addition of 0.5% cRBCs and incubation at room temperature for 40 min. The inhibition of hemagglutination at the highest serum dilution was considered as the HI titer of the serum.

### ELISPOT

Specific cellular immune responses were assessed by IFN-γ and IL-4 ELISPOT assays using mouse splenocytes. Assays were performed according to the instruction manual (U-CyTech, Netherlands). Briefly, ELISPOT 96-well plates Multiscreen Assay System (Millipore) were coated with anti-mouse IFN-γ and IL-4 capture Abs and incubated for 24 h at 4°C. On the following day, the plates were washed and blocked for 2 h with 1% BSA. 2 × 10^5 ^splenocytes from the immunized mice were added to each well and stimulated overnight at 37°C in 5% CO_2 _in the presence of RPMI 1640 (negative control), Con A (positive control), or 2 μg/ml purified HA-protein [33]. After 18 h of stimulation, the cells were washed and incubated for 1 h at 37°C with biotinylated detector antibodies (U-CyTech). The plates were washed, and streptavidin-HRP Conjugate (U-CyTech) was added to each well and incubated for 1 h at 37°C. The plate was washed, and AEC coloring system (U-CyTech) was added to each well. The plates were then rinsed with distilled water and dried at room temperature. Spots were counted by an automated ELISPOT Bioreader 4000 (Bio-Sys Limited Germany) [35]. The results were expressed as the number of spot forming cells (SFC) per 10^6 ^splenocytes cells in the ELISPOT experiment

### Virus titration

The bronchoalveolar wash was diluted 10-fold serially starting from a dilution of 1:10, inoculated on Madin Darby canine kidney (MDCK) cells, incubated at 37°C and examined for cytopathic effect 2 days later. The virus titer of each specimen, expressed as the 50% tissue culture infection dose (TCID_50_), was calculated by the Reed-Muench method [29]. The virus titer in each experimental group is represented by the mean ± SD of the virus titer per ml of specimens from all mice in each group.

### Statistical analysis

Statistical parameters (average values and standard deviations) were calculated using spss10.0 software. For comparisons of experimental groups, statistical analysis was performed using one-way ANOVA.spss for windows 10.0. *p *< 0.05 was considered significant. For survival, the probability was calculated by using Fisher's exact test, comparing the rate of survival in mice immunized with HA DNA to that of the control groups.

## Results

### Immunization with one HA DNA dose provided early protection in BALB/c mice against lethal dosage of H5N1 virus challenge

**Figure 1:**
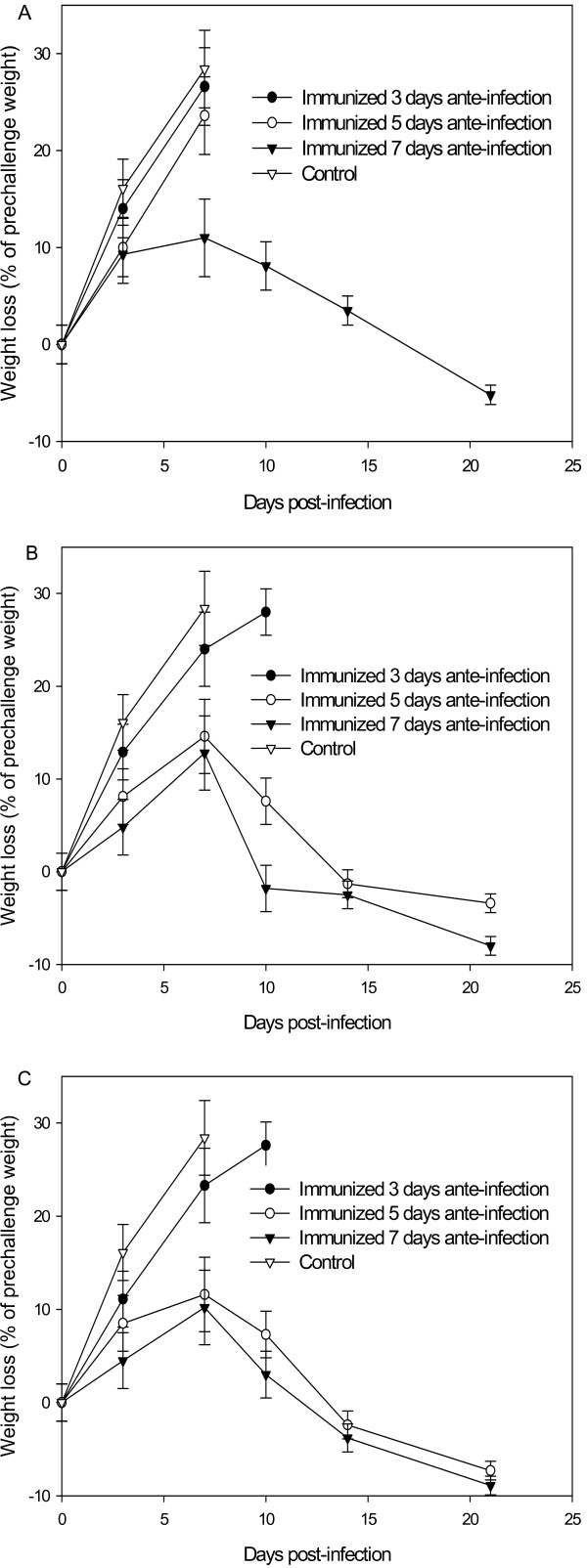
**Bodyweight changes after challenge**. Mice were immunized once with 10 μg (A), 50 μg (B) and 100 μg (C) of HA DNA 3, 5 and 7 days, respectively, before the lethal virus infection. Bodyweight changes were observed for 21 days after infection.

One hundred and sixty female BALB/c mice, at the age of 6–8 weeks, were divided into 4 groups. One group, containing 16 mice, was set up as control and the mice in this group were not immunized. The mice in the remaining three test groups (48 mice in each) were immunized at 3, 5 and 7 days, respectively, before the lethal viral challenge. To decide the injection dosage of the HA DNA, each test group was further divided into 3 subgroups, each containing 16 mice, and immunized with 10 μg, 50 μg or 100 μg of HA DNA, respectively. As for the challenge, all the mice, including the test and control groups, were infected by intranasal drip with a lethal dose (5 LD_50_) of homologous H5N1 virus. Three days after the infection, 4 mice from each subgroup and the control were taken out and killed for titration of residual lung virus. The rest of mice were observed for three weeks to evaluate the protective ability of the HA DNA by survival rate and bodyweight loss. The results are shown in Table 1 and Figure 1. Compared with the control, the mice immunized 3 days before the viral challenge, regardless of the injection dosage, had no significant difference in lung virus titers compared with the control mice. When mice were immunized 5 or 7 days before viral challenge, they had significantly lower lung virus titers than the control after lethal infection and the lung virus titer decreased as the injection dosage increased. The survival rate was also related to the immunization time and dosage. Mice immunized 3 days before viral challenge with HA DNA showed the survival rate of 0%, regardless of the injection dosage. Those immunized 5 days before viral challenge with HA DNA at the dosage of 10 μg, 50 μg and 100 μg showed the survival rate of 0%, 33% and 50%, respectively. When the mice were immunized 7 days before viral challenge, the mice had the survival rate of 50%, 83% and 100%, respectively, at the dosage of 10 μg, 50 μg and 100 μg. By contrast, the non-immunized mice all died within 7 days after the challenge and the lung virus titer reached as high as 10^6 ^TCID_50 _(Table 1). Bodyweight loss measurements also displayed the corresponding changes. The mice that gained better protection lost less bodyweight and therefore recuperated more quickly (Figure 1). These results showed that mice would gain partial protection 5 days after immunization, with more than 50 μg of HA DNA, and that, 7 days after the immunization, the protective ability would greatly increase.

**Table 1:** Protection of mice immunized with HA DNA vaccine against lethal H5N1 virus challenge^a^

		Lung virus titer^b^ (log_10 _TCID_50_/ml)			Survival rate (No. of survivors/no. tested)		
		
Plasmid DNA	Dose (μg)	3 days	5 days	7 days	3 days	5 days	7 days
HA DNA	10	6.25 ± 0.35	4.80 ± 0.28^c^	4.25 ± 0.35^c^	0/12	0/12	6/12
	50	5.75 ± 0.35	4.55 ± 0.07^c^	4.00 ± 0.70^c^	0/12	4/12	10/12^d^
	100	5.75 ± 0.35	4.55 ± 0.07^c^	3.25 ± 0.35^c^	0/12	6/12	12/12^d^
Control		6.25 ± 0.35			0/12		

^a ^Mice were immunized once with HA DNA 3, 5 and 7 days, respectively, before the challenge and at various dosages. Then all the mice were challenged under anesthesia with 5 LD_50 _of homologous A/Chicken/Henan/12/2004(H5N1) virus. Three days after the viral challenge, the mice were sacrificed. The trachea and lungs were taken out and washed twice by injecting 2 ml of PBS containing 0.1% BSA. The bronchoalveolar wash was used for virus titration after removing cellular debris by centrifugation. The survival of mice 3 weeks after the challenge was measured.

^b^Values represent means ± SD of 4 mice from each group.

^c ^Significantly different from the control groups (*p *< 0.05) by ANOVA.

^d ^Significantly different from the control groups (*p *< 0.05) by Fisher's exact test.

### Antibody response induced in mice at early stage when immunized once with various dosages of HA DNA

**Figure 2:**
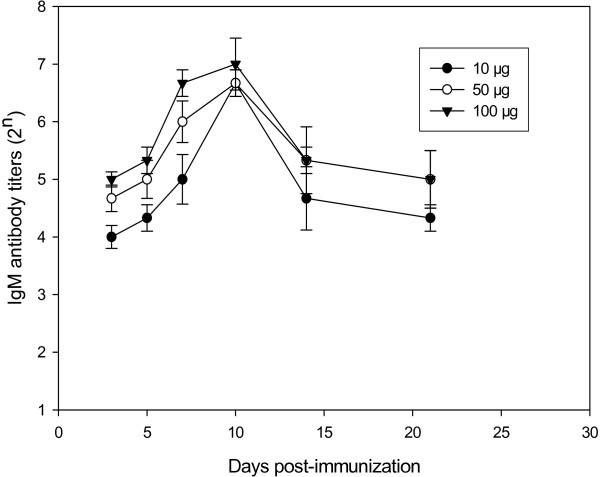
**IgM antibody titers in mice immunized once with various dosages of H5N1 virus HA DNA**. Twenty BALB/c mice aged 6–8 weeks were divided into 4 (n = 5) groups. One group of non-immunized mice was set up as a control. The mice in the three test groups were immunized once with 10 μg, 50 μg and 100 μg of HA DNA, respectively. Blood samples were collected on day 3, 5, 7, 10, 14, 21, and 28 after immunization and specific IgM antibodies were detected by ELISA. Ab-positive cut-off values were set as mean + 2 SD of non-immunized sera. An ELISA Ab titer was expressed as the highest serum dilution giving a positive reaction.

**Figure 3:**
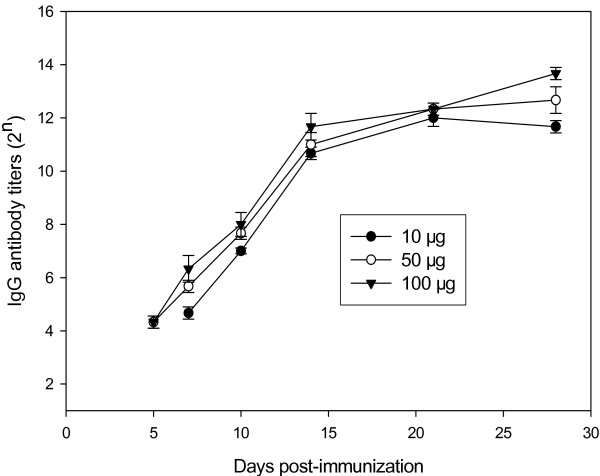
**IgG antibody titers in mice immunized once with various dosages of H5N1 virus HA DNA**. Twenty BALB/c mice aged 6–8 weeks were divided into 4 (n = 5) groups. One group of non-immunized mice was set up as a control. The mice in the three test groups were immunized once with 10 μg, 50 μg and 100 μg of HA DNA, respectively. Blood samples were collected on day 3, 5, 7, 10, 14, 21, and 28 after immunization and specific IgG antibodies were detected by ELISA. Ab-positive cut-off values were set as mean + 2 SD of non-immunized sera. An ELISA Ab titer was expressed as the highest serum dilution giving a positive reaction.

**Figure 4:**
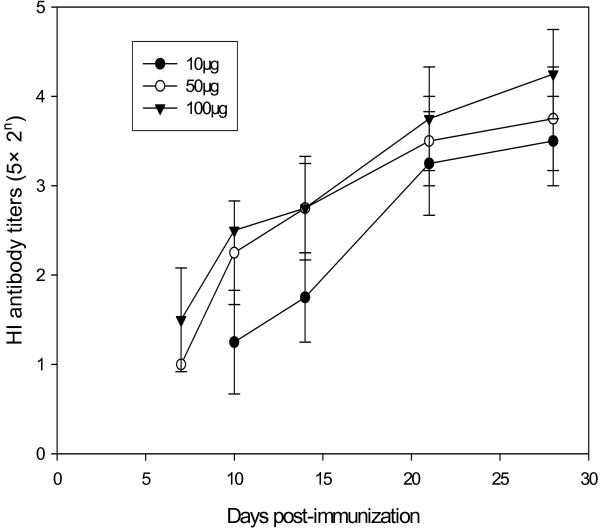
**HI titers in mice immunized once with various dosages of H5N1 virus HA DNA**. Blood samples collected on various days were detected for specific antibodies by hemagglutinin inhibition (HI) assay. No HI activity was detectable in non-immunized mice.

Twenty female BALB/c mice, at the age of 6–8 weeks, were divided into 4 groups, five mice each group. One group of non-immunized mice was set up as a control. The mice in the three test groups were immunized once with 10 μg, 50 μg and 100 μg of HA DNA, respectively. On day 3, 5, 7, 10, 14, 21, and 28 after the immunization, blood samples were collected from either the medial or lateral canthus of mice. Both IgM and IgG-specific antibodies were detected by ELISA. The results are shown in Figures 2 and 3. The results showed that immunization once with H5 virus HA DNA induced specific IgM and IgG antibodies in mice. The IgM antibody could be detected in all the immunized mice on the 3rd day after the immunization, reached the highest titer on the 10th day, and became undetectable on the 28th day. As for the IgG antibody, mice immunized with either 50 μg or 100 μg of HA DNA had the specific antibody detectable by ELISA on the 5th day, while those with 10 μg of HA DNA failed to develop detectable antibody until the 7th day. The IgG antibody titer increased gradually, and was maintained at a high level until the 28th day. Meanwhile, a high injection dosage of HA DNA induced relatively high titer of both IgM and IgG antibodies. The specific antibodies were further confirmed by HI assay (Figure 4). When mice were immunized with 10 μg of HA DNA, HI titers could not be detected until the 10th day after immunization. When mice were immunized with 50 μg or 100μg of HA DNA, HI titers could be detected on the 7th day. A high injection dosage of HA DNA also induced a relatively high HI titer.

### Cell-mediated immune response induced in mice at an early stage when immunized once with various dosages of HA DNA

**Figure 5:**
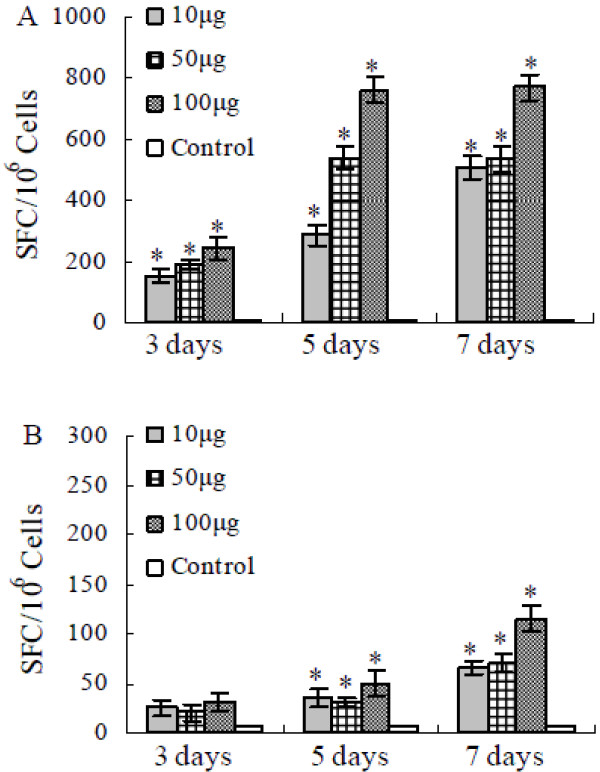
**HA protein-specific IFN-γ (A) and IL-4 (B) ELISPOT**. Splenocytes harvested from mice 3, 5 or 7 days after vaccination were stimulated with HA protein for 18 h and scored in ELISPOT assays for IFN-γ and IL-4 producing cells. The values represent the averages of triplicate wells of 4 mice, and are expressed as means ± SD. The results were expressed as the number of SFC per 10^6 ^input cells. * Significant difference (*p *< 0.05).

Forty female BALB/c mice, at the age of 6–8 weeks, were divided into 4 groups. One group, containing 4 mice, was set up as a control and the mice were not immunized. The three were test groups, each containing 12 mice, were immunized with 10 μg, 50 μg and 100 μg of HA DNA, respectively. Splenocytes were harvested from 4 immunized mice from each group at 3, 5 or 7 days after vaccination. The ELISPOT assay was used to assess the magnitudes of HA-specific IFN-γ (Th1) and IL-4 (Th2) T-cell responses after mice were vaccinated with HA DNA (Figure 5). The harvested splenocytes were stimulated with HA protein for 18 h and scored in ELISPOT assays for IFN-γ and IL-4 producing cells. As shown in Figure 5, only a low number of non-specific IFN-γ and IL-4 ELISPOTs were detected in the control groups (spots ≥ 10/10^6 ^cells). ELISPOT background counts in wells containing splenocytes in the absence of mitogens or nominal antigens were approximately the same as those in the control groups. The numbers of positive non-specific IFN-γ and IL-4 ELISPOTs (concanavalin restimulated) were high, up to 2000/10^6 ^cells (data not shown). Compared with the non-immunized control groups, significant numbers of HA-specific IFN-γ and IL-4 ELISPOTs were detected in all immunized groups (*p *< 0.05). A limited amount of IFN-γ and IL-4 secreting cells could be detected on the 3rd day after immunization. On both the 5th and the 7th days the IFN-γ secreting cells increased greatly. The group of mice immunized with 100 μg HA DNA elicited more IFN-γ and IL-4 secreting cells than the group of mice immunized with 10 μg or 50 μg DNA. Moreover, mice immunized once with HA DNA with dosages ranging from 10 μg to 100 μg raised significant amounts of IFN-γ-producing cells, but low levels of IL-4-producing cells, in response to the stimulation with H5 HA protein. All immunized groups raised 8 to 12 times more HA-specific IFN-γ-producing cells than IL-4-producing cells, suggesting a bias towards a Th1-type response. These results demonstrated that the HA DNA vaccine formulation had good immunogenicity, and induced an early cellular immune response (Figure 5).

## Discussion

To prepare for a pandemic influenza outbreak, any human isolate of a novel subtype should be considered as a potential hazard against humans and the feasibility of a vaccine production should be evaluated [36,37]. To control avian influenza, plasmid DNA vaccines have been considered as alternatives to inactivated vaccine. Many studies have shown that H1-, H3-, H5-, H7- and H9-DNA vaccines could protect mice and chickens against challenge with the homologous influenza virus [20-22,28,35].

In this study, BALB/c mice were immunized once by electroporation with HA DNA at different dosages. Three, 5 or 7 days after the immunization, the mice were challenged with a lethal dose (5 LD_50_) of H5N1 virus. The results showed that immunization once with 50 μg or 100 μg of HA DNA provided partial protection for mice against homologous H5N1 virus 5 days after immunization and that immunization once with 100 μg provided good protection against the H5N1 virus 7 days after immunization. We demonstrated that the immunity could be established shortly after the vaccination with a single dose of H5N1 DNA vaccine and that it afforded early protection against H5N1 virus in mice.

The breadth of the immune responses induced by the DNA vaccine included both cell-mediated and humoral immunities. Although most studies with HA DNA have reported that effective antibody responses were induced only after a booster immunization [38-40], a single dose of a DNA vaccine had also been proved effective [41]. Tsang et al recently demonstrated that a single intramuscular DNA vaccination, when combined with electroporation, significantly enhanced both the onset and duration of primary antibody response and possibly the duration of immune memory [42]. We obtained similar results in our previous experiments with H9 virus HA DNAs [27,28]. The present study demonstrated that a single dose of H5 virus HA DNA could induce specific IgM and IgG antibodies in mice (Figures 2 and 3). The IgM antibody, which appears prior to other kinds of antibodies during the immune response, was detectable in mice on the 3rd day after immunization, reached its highest value on the 10th day and then gradually disappeared. The IgG antibody appeared a little later. It was detectable on the 5th day and maintained a high level over a 4-week measurement. It indicated that a single dose of H5 virus HA DNA could induce a primary antibody response and that the level of the induced IgG antibody was long-lasting.

The cellular immune response is mediated by antigen specific CD4+ and CD8+ T cells. T cells cannot recognize free pathogens, but instead identify infected cells and exert effector functions, including direct cytotoxic effects and cytokine release [43,44]. It has been reported that muscle inoculation of DNA preferentially elicits a Th1-type response with relatively high CTL activity [45,46]. Our results demonstrated, besides circulating anti-influenza virus antibodies, a potent systemic cellular immune response following a single immunization of H5N1 HA DNA. The mice immunized once with HA DNA in dosages ranging from 10 μg to 100 μg raised significant amounts of IFN-γ-producing cells, but low levels of IL-4-producing cells, in response to the stimulation with H5-HA protein (Figure 5). These findings suggested that H5N1 DNA vaccine might be a potent inducer of systemic type 1 T cells, which were capable of mediating protection in the respiratory tract. The H5 HA DNA vaccine might, to some degree, induce a cytotoxic response in a naive population, although the cytotoxic functionality of the CD8+ T cells was not measured.

A series of human clinical trials with inactivated H5N1 vaccines demonstrated that inactivated H5N1 vaccines, with or without adjuvant, could elicit neutralization antibodies and cross-reactive neutralization antibodies to currently circulating variant H5N1 strains [47-50]. These clinical trials also showed us some unexpected findings. Nolan et al. recently demonstrated the better immune response for children compared to adults when they received two doses of inactivated, split H5N1 vaccine and the lower seroresponse to the H5N1 vaccines in those who had been previously vaccinated against seasonal influenza [49,50]. These findings suggested that pre-existing antibodies to human influenza viruses might influence the immunogenicity of H5N1 vaccines. It is worthy to know if these findings are only limited to inactivated vaccines. Furthermore, although the H5 HA DNA vaccine was shown in the present study to provide early protection against lethal challenge in mice model, how it would behave in a human body remains unknown.

## Conclusion

We have demonstrated that a single immunization of 100 μg of an H5 HA DNA vaccine, combined with electroporation, elicited both humoral and cellular immune responses, which provide mice the early protection against avian influenza H5N1 virus. It might be helpful for the ideal of providing rapid onset of protection against AIV infection with a "single shot" vaccine.

## Competing interests

The authors declare that they have no competing interests.

## Authors' contributions

LYZ did most of the experimental work and drafted the manuscript. FYW, ZDY and JJC participated in the analysis of humoral and cellular responses. HYC participated in the immunization of mice. ZC revised the manuscript for important intellectual content and gave final approval of the version to be published. All authors read and approved the final manuscript.

## Pre-publication history

The pre-publication history for this paper can be accessed here:

http://www.biomedcentral.com/1471-2334/9/17/prepub
